# Supplementary material for: Poorer verbal working memory for a second language selectively impacts academic achievement in university medical students
Source: PeerJ. 2013 Feb 12;1:e22. doi: 10.7717/peerj.22 (PMC3628612; doi:10.7717/peerj.22)
Supplement: Supplemental Information 1 [file peerj-01-22-s001.pdf]

MONASH University

EXPLANATORY STATEMENT

(This information sheet is for you to keep)

Project Title: English acquisition status and academic performance in MBBS students

Chief Investigators: Collette Mann
Ramesh Rajan
Benedict Canny
Anthony Luff

Project Co-ordinator: Jennifer Lindley

Introduction

My name is Collette Mann and I am conducting a research project with Ramesh Rajan, Ben Canny, Tony Luff and Jennifer Lindley in the Department of Physiology towards a PhD at Monash University. This means that I will be writing a thesis, which is the equivalent of a short book.

Research Background

The aim of this study is to understand how the age that one acquires a second language, (in this case English), may influence academic performance in students. We also want to investigate if the language acquisition age impacts on how English speech is understood when there is background noise. It is widely reported in other countries that academic performance can be affected by a number of factors, including the age of when English is learnt and we would like to establish if these observations are also noted in Australian students. A very important outcome of our study could be to design better strategies to support learning in any student who has difficulty in understanding speech in background noise (e.g., because of hearing loss, specific language impairment, etc).

For our study we have chosen students from the MBBS course, such as yourself, as you have strong academic records and, at least, a good level of English proficiency. Both of these criteria help in eliminating variation in our data.

We would like to request your voluntary participation in this study, whether you speak English as a first language, as a second or third language, or even if you speak only English.

Conditions
If you agree to participate in this study, you will be asked to carry out two tasks and consent to our conducting analysis in a third stage where we will compare data from the initial two tasks with your academic performance data (coded to ensure confidentiality). The tasks you will be asked to carry out are:

1. To fill out a questionnaire, which asks about when you acquired English, your language skills, the other languages you speak and your proficiency in these languages relative to English, your musical skills, learning style, etc.
2. Participate in a brief series of hearing tests where your ability to identify a standard set of sentences in the presence of background noise (speech-in-noise tests) will be assessed in a single session that will last for about 30 minutes in total. We will also measure your hearing sensitivity using a standard test called audiometry.

Finally, so that we can determine how language acquisition is associated with learning outcomes, your data for the questionnaire and your results for speech-in-noise tests will be matched with your academic records that are already collected by the faculty.

Risks
The hearing tests are not painful or dangerous. The audiometry test is a standard audiological test of hearing sensitivity. In the speech-in-noise tests, the noises used mimic everyday noises and are presented at comfortable listening levels and are not in any way injurious or painful. So, there are no risks involved. If we find that you show any signs of hearing loss as defined by standard audiological criteria, then we will advise you to see a doctor and get a referral to an audiologist.

Confidentiality
Please note that your confidentiality will be protected at all times. All your data will be coded so that none of your teachers or any of the experimenters involved in analysis of the data will be able to identify you. Only the project co-ordinator, Ms Jennifer Lindley, will have access to any identifiable data, but she will not have access to the analyses so that she will not be able to link any personal data to analyses of performance outcomes. Further, no findings, which could identify any individual participant, will be published.

All data will be stored for at least five years on University premises, in accordance with University ethics and privacy guidelines. You may access the information that you provide at any time by contacting any one of the project staff.

You should also be aware that research data for this study might be provided to other researchers for future research, but only as group data that cannot be identified to any individual person.

MONASH University

Can I withdraw from the research?
Being in this study is voluntary and you are under no obligation to consent to participation. However, if you do consent to participate, **you may withdraw your consent at any time without any penalty to you.**

If you would like to be informed of the aggregate research finding or if you have any questions and would like further clarification of any aspect of this study, please feel free to ask me. My contact details are:

Collette Mann
ph: 9905 8003 email: Collette.Mann@med.monash.edu.au

If you would like to contact the **researchers** about any aspect of this study, please contact the Chief Investigator:	If you have a **complaint** concerning the manner in which this research CF08/2667 - 2008001361 is being conducted, please contact:
A/Prof Ramesh Rajan<br>ph: 9905 2525<br>email: Ramesh.Rajan@med.monash.edu.au	Human Ethics Officer<br>Standing Committee on Ethics in Research Involving Humans (SCERH)<br>Building 3e Room 111<br>Research Office<br>Monash University VIC 3800<br>Tel: +61 3 9905 2052 Fax: +61 3 9905 1420 Email: scerh@adm.monash.edu.au

Thank you,

Collette Mann

MONASH University

DISCLOSURE AND CONSENT FORM

Project Title: *English acquisition status and academic performance in MBBS students*

NOTE: This consent form will remain with the Monash University researcher for their records

I state that I am currently enrolled in the MBBS (Bachelor of Medicine, Bachelor of Surgery) course at Monash University and agree to take part in the Monash University research project specified above. I have had the project explained to me, and I have read the Explanatory Statement, which I keep for my records. I understand that agreeing to take part means that I am willing to:

- complete questionnaires asking me about my personal details and language skills.
- attend a single session of 6 hearing tests that takes ~30 minutes in total arranged for a mutually convenient time with the researchers.
- allow access to my academic records for the purpose of data analysis.

I understand that my participation is voluntary, that I can choose not to participate in part or all of the project, and that I can withdraw at any stage of the project without being penalised or disadvantaged in any way.

I understand that the audiometry tests are not dangerous, but mimic sounds common in everyday life and that if hearing impairment is indicated that the researchers will advise me to consult with my GP to obtain a referral to an audiologist.

I understand that any data that the researcher extracts from the questionnaire /survey for use in reports or published findings will not, under any circumstances, contain names or identifying characteristics.

I understand that I can access my information upon request.

I understand that data collected will be stored for a period of at least 5 years in accordance with the University ethics and privacy guidelines.

I agree that research data collected for the study may be published or provided to other researchers for future research on the condition that anonymity is preserved and that I cannot be identified.

Participant's name ____

Signature __________________________________**Date** _____/_____/______
